# Influence of Admission Time on Health Care Quality and Utilization in Patients with Stroke: Analysis for a Possible July Effect and Weekend Effect

**DOI:** 10.3390/ijerph182312362

**Published:** 2021-11-24

**Authors:** Chun-Yi Liu, Pei-Tseng Kung, Hui-Yun Chang, Yueh-Han Hsu, Wen-Chen Tsai

**Affiliations:** 1 Department of Health Services Administration, China Medical University, Taichung 406040, Taiwan; lcy@mail.cmuh.org.tw (C.-Y.L.); a6636a@gmail.com (H.-Y.C.); 2 Department of Education, China Medical University Hospital, Taichung 404332, Taiwan; 3 Department of Healthcare Administration, Asia University, Taichung 413305, Taiwan; ptkung@asia.edu.tw; 4 Department of Medical Research, China Medical University Hospital, China Medical University, Taichung 404332, Taiwan; 5 Division of Nephrology, Department of Internal Medicine, Ditmansion Medical Foundation Chia-Yi Christian Hospital, Chia-Yi 600566, Taiwan; cych07023@gmail.com; 6 Department of Medical Research, Ditmansion Medical Foundation Chia-Yi Christian Hospital, Chia-Yi 600566, Taiwan; 7 Department of Nursing, Min-Hwei College of Health Care Management, Tainan 736302, Taiwan

**Keywords:** July effect, weekend effect, quality, utilization, stroke

## Abstract

(1) Purpose: Undesirable health care outcomes could conceivably increase as a result of the entry of new, less experienced health care personnel into patient care during the month of July (the July effect) or as a result of the less balanced allocation of health care resources on weekends (the weekend effect). Whether these two effects were present in Taiwan’s National Health Insurance (NHI) system was investigated. (2) Methods: The current study data were acquired from the NHI Research Database. The research sample comprised ≥18-year-old patients diagnosed as having a stroke for the first time from 1 January 2006 to 30 September 2012. The mortality rate within 30 days after hospitalization and readmission rate within 14 days after hospital discharge were used as health care quality indicators, whereas health care utilization indicators were the total length and cost of initial hospitalization. (3) Results: The results revealed no sample-wide July effect with regard to the four indicators among patients with stroke. However, an unexpected July effect was present among in-patients in regional and public hospitals, in which the total lengths and costs of initial hospitalization for non-July admissions were higher than those for July admissions. Furthermore, the total hospitalization length for weekend admissions was 1.06–1.07 times higher than that for non-weekend admissions; the total hospitalization length for weekend admissions was also higher than that for weekday admissions during non-July months. Thus, weekend admission did not affect the health care quality of patients with stroke but extended their total hospitalization length. (4) Conclusions: Consistent with the NHI’s general effectiveness in ensuring fair, universally accessible, and high-quality health care services in Taiwan, the health care quality of patients examined in this study did not vary significantly overall between July and non-July months. However, a longer hospitalization length was observed for weekend admissions, possibly due to limitations in personnel and resource allocations during weekends. These results highlight the health care efficiency of hospitals during weekends as an area for further improvement.

## 1. Introduction

In July, hospitals tend to receive newly recruited health care personnel or those with less experience, as well as interns and residents who have just graduated from medical schools; this might exert a negative effect on the treatment outcomes of patients, called the “July effect” [1]. This effect is generally associated with erroneous decisions made by less experienced staff members or those demonstrating poor efficiency. During a cohort turnover in a teaching hospital, numerous new recruits enter the workplace at the same time, while relatively more experienced staff members might resign, resulting in the loss of tacit knowledge. This suddenly reduces the mean years of experience of staff members and undermines the previously established teamwork structure, thereby reducing overall productivity. Cohort turnover occurs once each year and affects over 100,000 hospital staff members in the United States and 32,000 in Europe [2]. The undesirable situation associated with this phenomenon is referred to as the “August killing season” in the United Kingdom and the July phenomenon or July effect in the United States [2,3].

Medical education is a core mission of teaching hospitals and a crucial determinant affecting the health care services provided by interns and residents. When residents first enter a hospital, they learn to become full-fledged physicians and care for patients; however, they may also make mistakes during this learning period [4]. Medical educators, news media, and the public have noted that health care errors by hospital staff members tend to increase in July; this repeating cycle has led to the public believing that they should try to stay healthy during this month [5]. Although some studies have not verified the presence of the July effect [1,6], one study has observed poor medical outcomes during the months affected by the July effect [3]. However, whether adverse events increase or service quality worsens during the transition period affected by the July effect remains uncertain, potentially because previous studies used relatively small sample sizes, an insufficient case mix, and less common medical outcomes (e.g., in-hospital mortality) and did not collect supervision-related information provided by residents [3,5,6].

The “weekend effect” is a unique phenomenon in the medical service industry. For patients hospitalized during weekends or holidays, medical outcomes may be poorer. This is because during weekends or holidays, the allocation of medical personnel and completeness of supervision mechanism are less than that during weekdays. Additional medical resources may be used when patients are hospitalized at this time [7,8,9]. The prognoses of acute patients admitted through the emergency department on weekends are poorer than those of such patients admitted on weekdays. A reason for this undesirable outcome may be the delay from symptom onset to diagnosis, intervention, and treatment. Treatment delay can increase complication, hospitalization length, and medical cost [10]. Among patients with myocardial infarction, those admitted on weekends have a higher mortality rate than those admitted on weekdays. In addition, patients with weekend admissions have a lower probability of receiving invasive cardiac surgery than those with weekday admissions [11]. For the general public, the weekend effect is the discrepancy in the medical outcomes between weekend and weekday admissions. The causes of the weekend effect include a lack of medical personnel and senior staff members, a decreased number of services delivered for a clinical subspecialty, and a decreased execution rate of invasive treatments on weekends [12].

On the basis of the aforementioned description, the July effect refers to undesirable health care outcomes due to insufficient clinical experience among health care personnel, whereas the weekend effect may be caused by imbalanced health care resource allocation. This study aimed to explore the July effect and weekend effect, and their combined influence on the health care quality and utilization in patients with stroke—with a mortality rate within 30 days after hospitalization and readmission rate within 14 days after hospital discharge as health care quality indicators and the total length and cost of initial hospitalization as health care utilization indicators.

According to the World Health Organization website and official Department of Statistics of the Ministry of Health and Welfare data, cardiovascular disease is the primary cause of mortality among patients with chronic noncommunicable diseases globally. Cardiovascular diseases are caused by heart and vascular diseases, including coronary heart disease (heart attack), cerebrovascular disease (stroke), high blood pressure (hypertension), peripheral artery disease, rheumatic heart valve disease, etc. The impact of stroke depends on the severity of the brain injury, and a very serious stroke can cause sudden death. For carting hospitalized stroke patients, hospitals need to provide emergency examinations and medical treatment. More medical resources and manpower are needed to support the treatment. The promptness of treatment and arrangement of medical manpower are important issues for hospitals and patients.

Cardiovascular disease substantially affects people’s health worldwide. Hence, according to the level of influence of cardiovascular disease and the research targets adopted in previous studies, the present study targets patients with stroke as the study population and discusses the medical quality and medical utilization of the treatment for cerebrovascular diseases (stroke).

## 2. Materials and Methods

### 2.1. Data Source and Study Population

The study data were acquired from National Health Insurance (NHI) Research Database (NHIRD), overseen by the National Health Research Institute of Taiwan. In particular, this study used the Longitudinal Health Insurance Database, which contained the claims data of two million people, randomly sampled from the 23 million Taiwan residents in 2000 and 2005, respectively. No significant differences were observed between the total population of Taiwan and the samples, in terms of age, sex, and average insurance cost, thus verifying that the file data were representative of all 23 million Taiwan residents (http://nhird.nhri.org.tw/date_cohort.html, accessed on 11 March 2017). In the NHIRD, personal data are encrypted. The sampling period was from 1 January 2006 to 30 September 2012. This study was approved by an institutional review board (IRB number: CMUH104-REC3-099).

Patients, aged ≥18 years, diagnosed as having stroke for the first time during the sampling period were included. The diagnoses were confirmed using the International Classification of Diseases, Ninth Revision, Clinical Modification (ICD-9-CM) codes for stroke (431, 432×, 433 × 1, 434 × 1, 435×, and 436). These study targeted patients whose primary diagnosis code contains one of these numbers. If an in-patient was transferred from the emergency department, the day the patient arrived in the emergency department was considered the initial day of hospitalization.

### 2.2. Research Variables

The dependent variables of this study were divided into two groups:

1. Health care quality indicators (i.e., mortality within 30 days after hospitalization and readmission within 14 days after hospital discharge).
2. Health care utilization indicators (i.e., total length and cost of initial hospitalization).

The independent variables of this study were divided into four groups:

1. Patient characteristics (i.e., sex, age (18–44, 45–54, 55–64, 65–74, and ≥75 years old), urbanization level of the residential area (divided into seven levels, with the first and seventh levels denoting highly urbanized and remote regions, respectively), insured salary based on the categorization defined by the National Health Insurance (NHI) (≤NT$17,280, NT$17,281–NT$22,800, NT$22,801–NT$28,800, NT$28,801–NT$36,300, NT$36,301–NT$45,800, NT$45,801–NT$57,800, and ≥NT$57,801), Charlson comorbidity index (CCI; developed by Deyo et al. [13] in 1992; divided into five levels of 0, 1–3, 4–6, 7–9, and ≥10), and whether they underwent any surgery).
2. Hospital characteristics (i.e., hospital level (medical center or regional hospital), ownership (public or private hospital), urbanization level of hospital location (divided into seven levels, with first and seventh levels denoting highly urbanized and remote regions, respectively), total number of stroke patients treated per year (>75th, 25–75th, and <25th percentiles denoted high, medium, and low patient numbers, respectively), and total number of neurologists and neurosurgeons).
3. Specialist characteristics (i.e., number of patients treated per year (>75th, 25–75th, and <25th percentiles denoted high, medium, and low patient numbers, respectively)).
4. Hospital admission time (i.e., July weekday admissions (the July effect), non-July weekend admissions (the weekend effect), July weekend admissions (combined influence of the July effect and weekend effect), and non-July weekday admissions).

### 2.3. Statistical Analysis

Categorical variables were presented as frequency and percentile, whereas continuous variables were presented as mean and standard deviation (mean ± SD) for descriptive statistics. Inferential statistics comparing the differences in health care quality indicators of patients included chi-square test and *t* tests.

Covariates used in this study, such as patient factors, specialist factors, hospital factors, and patient admission times, were further controlled and analyzed using multivariate regression models with the generalized estimating equation approach (GEE approach). To examine the relationships between different independent and dependent variables as well as changes in these relationships, dichotomous dependent variables (i.e., mortality rate within 30 days after hospitalization and readmission rate within 14 days of hospital discharge) were subjected to logistic regression analysis, whereas continuous dependent variables (i.e., total length and cost of initial hospitalization) were subjected to multiple regression analysis. Because total length and cost of initial hospitalization do not follow a normal distribution, these two variables were converted to their natural logarithmic values before analysis. After controlling for covariates, this study analyzed the influence of the July effect and weekend effect on the health care quality and utilization indicators. Moreover, we included hospital levels (medical center or regional hospital), ownership (public or private), and urbanization levels of hospital locations (Levels 1–2 and 3–7) in the analysis. The research design of this study has taken into account the hospital cluster effect, indicating that the effects of patients receiving treatment are similar in the same hospital. Therefore, the above statistical analysis utilized the generalized estimating equation approach (GEE approach) for multivariate regression models.

This study further conducted the examination of the interaction relationship between the variables of hospital characteristics (hospital levels, hospital ownership, and urbanization levels of hospital location) and July/weekend effects on health care quality indicators and health care utilization indicators.

All statistical analysis was performed using SAS 9.4 (SAS Institute Inc., Cary, NC, USA).

## 3. Results

### 3.1. Sociodemographic Characteristics

This study identified 24,357 patients with stroke between 1 January 2006 and 30 September 2012. Of these, 9764 (40.09%) were female, and 14,593 (59.91%) were male. Most patients were aged ≥75 years (8592; 35.38%), were living in level-2 urbanization areas (7162; 29.4%), and had undergone surgery (16,467; 67.61%). The number of patients receiving treatment in regional hospitals (14,538; 59.69%) was higher than that in medical centers (9819; 40.31%); similarly, this number was higher in private hospitals (17,526; 71.95%) than in public hospitals (6831; 28.05%). Most hospitals and physicians treated a high number of patients (20,096; 82.51% and 15,115; 62.06%, respectively). Most patients were admitted in months other than July (22,341; 91.72%), while only 2016 were admitted in July (8.28%). Most patients were admitted on weekdays (17,437; 71.59%), while only 6920 were admitted on weekends (28.41%). Among the four admission times, most patients constituted non-July weekday admissions (13,023; 53.47%). Regarding the health care quality indicators, most patients survived within 30 days after hospitalization (22,754; 93.42%), while 1603 (6.58%) did not. Most patients were not readmitted within 14 after hospital discharge (23,372; 95.96%), while 985 (4.04%) were. Regarding the health care utilization indicators, the mean ± SD total length and cost of initial hospitalization for 24,357 patients were 14.19 ± 24.69 days and NT$93,319 ± NT$188,233, respectively (Table 1).

### 3.2. July Effect and Weekend Effect

Logistic regression was used to analyze the variables including patient factors, specialist factors, hospital factors, and admission times. In patients who died within 30 days after hospitalization, Table 2 presents that the mortality rate for non-July weekday admissions (reference group) did not significantly differ from that of July weekday, non-July weekend, and July weekend admissions (*p* > 0.05). In addition, in patients who were readmitted within 14 days after hospital discharge, the readmission rate for non-July weekday admissions (reference group) did not significantly differ from that of July weekday, non-July weekend, and July weekend admissions (*p* > 0.05). These results indicated that neither the July effect nor weekend effect was observed in the health care quality indicators when patients with stroke received treatments at medical institutions. However, the risk of readmission within 14 days after hospital discharge increased annually from 2006 to 2012. In 2012, this risk was 2.05 (95% confidence interval (CI), 1.60–2.64) times higher than that in 2006, and significant differences were observed from 2008 to 2012 (*p* < 0.05).

Table 3 demonstrates that the total length and cost of initial hospitalization for non-July weekday admissions (reference group) did not significantly differ from those of July weekday, non-July weekend, and July weekend admissions (*p* > 0.05). This result indicated that neither the July effect nor the weekend effect was observed with regard to the health care quality indicators when patients with stroke received treatments at medical institutions.

### 3.3. Stratified Analysis on Different Hospital Characteristics Levels

On the basis of the results in Table 1, Table 2 and Table 3, this study determined that the July effect and weekend effect had nonsignificant influence on health care indicators. This study further examined the interaction relationship between hospital characteristics (hospital levels, hospital ownership, and urbanization levels of hospital location) and July/weekend admission on health care quality indicators and health care utilization indicators. We found no interaction effect between hospital characteristics and July/weekend admission on health care quality indicators (*p* > 0.05). Nevertheless, the interaction relationship existed between hospital characteristics and July/weekend admission on health care utilization indicators (*p* < 0.05). However, there was no interaction effect between hospital level and July/weekend admission on the total cost of initial hospitalization. In addition, there was no interaction effect between hospital ownership and July/weekend admission. The study did not further perform the stratified analysis for hospital level and ownership on the total cost of initial hospitalization.

Therefore, this study further performed a stratified analysis by dividing hospital characteristics (i.e., hospital levels, hospital ownership, and urbanization levels of hospital locations) into different levels to examine whether these characteristics influenced the health care utilization indicators. The hospital characteristics were first divided into hospital level (medical center or regional hospital), ownership (public or private hospital, the latter of which included private hospitals and foundation hospitals), and urbanization level of hospital locations (levels 1–2 and levels 3–7). Accordingly, this study examined how the stratified hospital characteristics affected health care utilization indicators (i.e., total length and cost of initial hospitalization). Table 4 and Table 5 present the stratified analysis results.

Table 4 shows that in the hierarchical analysis of health care utilization indicators, the medical center did not have the July effect, but the regional hospitals had the July effect. The stroke patients in the regional hospitals had a shorter stay and less medical expenses in July. However, in terms of the weekend effect, both medical centers and regional hospitals had a relatively longer total length of initial hospitalization. The July effect appeared in public hospitals, but it did not appear in private hospitals. The stroke patients in public hospitals had a shorter hospital stay and used less medical expenses in July. However, in terms of the weekend effect, both public hospitals and private hospitals had a relatively longer total length of initial hospitalization. The weekend effect occurs for hospitals in the locations of urbanization level 1–2, but it does not occur for hospitals in the locations of urbanization level 3–7.

The total hospitalization length differed significantly (*p* < 0.05): In medical centers, the hospitalization length for weekend admissions was 1.07 times that for weekday admissions. In regional hospitals, the hospitalization length for July admissions was 0.94 times that for non-July admissions, and the hospitalization length for weekend admissions was 1.06 times that for weekday admissions. In public hospitals, the hospitalization length for July admissions was 0.92 times that for non-July admissions, and the hospitalization length for weekend admissions was 1.07 times that for weekday admissions. In private hospitals, the hospitalization length for weekend admissions was 1.06 times that for weekday admissions. In hospitals located in level 1–2 urbanization regions, the hospitalization length for weekend admissions was 1.07 times that for weekday admissions. Regarding the total cost of initial hospitalization, the following results were obtained: In regional hospitals, the hospitalization cost for July admissions was 0.90 times that for non-July admissions. In public hospitals, the hospitalization cost for July admissions was 0.88 times that for non-July admissions. In hospitals located in level 1–2 urbanization regions, the hospitalization cost for weekend admissions was 1.03 times that for weekday admissions. In hospitals located in level 3–7 urbanization regions, the July hospitalization admissions were 0.90 times that for non-July admissions.

Table 5 indicates that in the hierarchical analysis of health care utilization indicators, both medical centers and regional hospitals had relatively longer total length of initial hospitalization, resulting in the weekend effect. Both public and private hospitals had relatively longer total length of initial hospitalization, resulting in the weekend effect. Hospitals in the location of urbanization level 1–2 have July and weekend effects, but hospitals in the location of urbanization level 3–7 do not have July and weekend effects.

The total length of initial hospitalization differed significantly in relation to the following variables (*p* < 0.05): In medical centers, the hospitalization length for non-July weekend admissions was 1.07 times that for non-July weekday admissions. In regional hospitals, the hospitalization length for non-July weekend admissions was 1.07 times that for non-July weekday admissions. In public hospitals, the hospitalization length for non-July weekend admissions was 1.06 times that for non-July weekday admissions. In private hospitals, the hospitalization length for non-July weekend admissions was 1.07 times that for non-July weekday admissions. In hospitals located in level 1–2 urbanization regions, the hospitalization length for non-July weekend admissions was 1.07 times that for non-July weekday admissions. Finally, in hospitals located in level 1–2 urbanization regions, the total hospitalization cost of July weekday admissions was 0.96 times that for non-July weekday admissions, reaching a significant difference (*p* < 0.05). The aforementioned results indicated the presence of the weekend effect with regard to the total hospitalization length of patients with stroke.

## 4. Discussion

### 4.1. Influence of July Effect on Health Care Quality Indicators

Several studies have posited that in July when the number of new recruits in hospitals increases, the July effect is observable in health care services received by patients [1,3,14,15]. Studies have also indicated the absence of the July effect [16,17,18,19,20]. Studies have also reported the absence of the July effect was observed in the hospitalization mortality rate, surgical mortality rate, surgical complication rate, mortality rate within 30 days following surgery, unexpected hospital discharge rate, adverse event incidence rate, and medical complication rate [6,21,22,23,24,25]. Another study revealed that compared with that in other months, in the months when new residents first assumed their positions in a hospital, the mortality rate of patients in the surgery department was significantly higher, but the total mortality rate, mortality rate of patients in the internal medicine department, and mortality rate of patients in the ICU did not differ significantly [26]. The present study verified that among July and non-July admissions, no July effect was observed in the mortality rate within 30 days after hospitalization and readmission rate within 14 days after hospital discharge; these results concur with those of the aforementioned studies.

This study performed a stratified analysis on hospital characteristics by classifying hospitals into medical centers, regional hospitals, public hospitals, private hospitals, and hospitals located in level 1–2 or 3–7 urbanization regions and verified whether the July effect was present in these hospitals. The results revealed that no July effect was observed in the mortality rate within 30 days after hospitalization or 14 days after hospital discharge of patients living in medical centers, where teaching activities were more frequent, and regional hospitals, where teaching activities were less frequent. Shahian et al. [27] determined the mortality rate within 30 days after hospitalization of patients with acute myocardial infarction (AMI), congestive cardiac failure, and pneumonia in the United States; the authors discovered that the mortality rate of patients in hospitals with more frequent teaching activities was lower than that of patients in hospitals with less frequent teaching activities. Another study conducted in the United States reported that the mortality rate of patients receiving colon resection in teaching hospitals was 1.14 times that of patients in nonteaching hospitals [23]. The results of the present study differ from those of the aforementioned studies because patients in Taiwan are mainly managed by attending physicians, and clinical tasks performed by new residents are under the supervision of more experienced physicians, meaning that new residents do not perform health care tasks independently. Therefore, in the months when the number of newly recruited health care personnel increases, the health care quality of hospitals is not affected.

### 4.2. Influence of July Effect on Health Care Utilization Indicators

Several studies have posited that the mean hospitalization length in July did not significantly differ from that in other months [6,17,19]. The results of the present study concur with those of the aforementioned studies because the hospitalization length for July admissions with stroke was 0.97 times that for non-July admissions; no significant differences were observed (*p* > 0.05). This study also determined that the total hospitalization length for July admissions was 0.94 times that of non-July admissions in regional hospitals with less frequent teaching activities. The total hospitalization length for July admissions was 0.92 times that of non-July admissions in public hospitals. July effect was observed in patients receiving treatments at regional and public hospitals, but the total hospitalization length was shorter for July admissions. Similar results were observed regarding the total cost of initial hospitalization. In regional hospitals, the total hospitalization cost for July admissions was 0.90 times that of non-July admissions. In public hospitals, the total hospitalization cost for July admissions was 0.88 times that of non-July admissions. In hospitals located in level 3–7 urbanization regions, the total hospitalization cost for July admissions was 0.90 times that of non-July admissions. The July effect was observed in patients receiving treatments at regional and public hospitals located in level 3–7 urbanization regions, but the total hospitalization cost was higher for non-July admissions. Some studies have indicated that many Chinese people believe that it is ominous to undergo surgery in Chinese lunar July; these studies have noted that the number of surgery and mortality rate during Chinese lunar July is lower than that in other months [28,29]. In the present study, the total length and cost of initial hospitalization of admissions in Chinese lunar July were shorter and lower than those for non-July admissions, respectively. The reason may be that most days in July in the Western calendar actually correspond to Chinese lunar June. Most people are reluctant to be hospitalized in Chinese lunar July and prefer to be discharged from hospitals during this period, if possible. Concurring with the traditional beliefs of Chinese people, the present study observed that the total length and cost of initial hospitalization for July admissions were shorter and lower than those for non-July admissions, respectively.

### 4.3. Influence of Weekend Effect on Health Care Quality Indicators

Studies have indicated that the health care quality is influenced by weekend admissions. For instance, the risks of major adverse cardiac events in patients with AMI or heart diseases within 30 days following weekend admissions were 2.1 times that of patients admitted during weekdays [30]. The mortality rate within 30 days for weekend admissions was higher than that for weekday admissions [31,32,33,34,35,36,37]. The door-to-balloon time was longer in weekend and night-time admissions [34], in which the incidence rate of >120-min door-to-balloon time for weekend admissions (41.5%) was higher than that for weekday admissions (27.7%; *p* < 0.05) [37].

Some studies have explored the mortality rate of patients with stroke. In their US study, Reeves et al. [38] determined that the mortality rate of patients with ischemic or hemorrhagic stroke was higher when admitted during weekends than during weekdays. In their study in Canada, Saposnik et al. [36] determined that the mortality rate of patients with stroke for weekend admissions was 17% higher than that for weekday admissions and that the early mortality rate for weekend admissions was higher than that for weekday admissions. Studies on the other types of patients with weekend admissions have also determined that the mortality rate for weekend admissions, mortality rate within 48 h after hospitalization, surgical intervention rate, incidence rate of adverse events, and mortality rate in emergency surgery were higher than patients admitted during weekdays [39,40,41,42]. A UK study revealed that the total risk of death in patients admitted through the emergency department on weekends was 10% higher than those admitted on weekdays [43] and that the mortality rate was 16% higher on Sunday than on Wednesday [44]. However, a US study revealed that the mortality rate of patients with trauma was lower in weekend admissions than in weekday admissions (odds ratio, 0.89; 95% CI, 0.81–0.97) [45].

Numerous studies on the weekend and non-weekend admissions of patients with AMI or heart disease have reported the absence of the weekend effect [34,46,47,48,49,50]. In patients with stroke, no differences were observed in the mortality rate and other clinical indicators between weekend or non-weekend admissions [51], and no differences were observed in the mortality rate within 7 days after hospitalization, myocardial infarction rate, overall mortality rate, and complication rate [52]. The present study results revealed that the weekend effect was not observed in the health care quality indicators (i.e., mortality rate within 30 days after hospitalization and readmission within 14 days after hospital discharge). The results of the present study concur with those in the aforementioned studies, which did not observe significant differences between weekend and non-weekend admissions. The reason for this may be that the NHI system facilitates a fair and universal health care environment in Taiwan. The system now covers 99.9% of Taiwan’s population and has service contracts with 93% of Taiwan’s hospitals and clinics. To ensure that Taiwan people receive complete medical services, the NHI allows people to select hospitals according to their needs and provides excellent and convenient health care services. According to the results of this study, the complete NHI system and its benefit package have increased the stability of Taiwan’s health care system and structure. In Taiwan, hospitals also provide universal, consistent, continuous, and complete health care services through their excellent health care quality management. Hence, no unfavorable influences were observed in health care quality.

The risk of readmission within 14 days after hospital discharge increased yearly from 2006 to 2012. Therefore, hospitals need to further improve their health care quality.

### 4.4. Influence of Weekend Effect on Health Care Utilization Indicators

Some studies have reported the absence of differences in the total hospitalization length or cost between weekend and non-weekend admissions [10,53,54]. By contrast, some studies have revealed that the weekend effect was observed in hospitalization length [39,41,42,45,55]. This study found that whether it is a medical center or a regional hospital, a public hospital, or a private hospital, the weekend effect appeared in the total length of initial hospitalization. The hospital in the location of urbanization level 1–2 also has the weekend effect. The total length of initial hospitalization is relatively longer for patients admitted to the hospital on weekends.

The results of the present study determined that weekend admissions did not influence the health care quality but extended the total length of initial hospitalization and that the weekend effect was observed in the total length of initial hospitalization but was not significantly observable in the total cost of initial hospitalization. The hospitalization length for weekend admissions was longer than that for weekday admissions because of limitations in personnel and resource allocations on the weekend, during which the inspection, examination, and surgery provided are not as complete and standardized as those on weekdays.

In short, this study initially expected that the influence of July effect, weekend effect, or their combination would be observable even in Taiwan where the NHI is highly prevalent. However, the results indicated that these effects were not very obvious. Because the NHI enables a fair, prevalent, high-quality, consistent, and excellent health care environment in Taiwan, the health care quality of patients examined in this study did not vary significantly with different admission times. However, this study found that the hospitalization length for weekend admissions was significantly longer than that for non-weekend admissions. The health care efficiency of hospitals during the weekend required improvement.

## 5. Conclusions

In patients with stroke admitted in July and in months other than July, no July effect was observed with respect to the health care indicators, including the mortality rate within 30 days after hospitalization and readmission rate within 14 days after hospital discharge. In patients with stroke and receiving treatments at regional or public hospitals, the total hospitalization length for July admissions was shorter than that for non-July admissions. Hence, the July effect was observed in patients receiving treatments at regional and public hospitals, but the total hospitalization length for non-July admissions was longer than that for July admissions. Similar results were determined in the total cost of initial hospitalization. The total cost of initial hospitalization for July admissions was lower than that for non-July admissions at regional hospitals, public hospitals, and hospital located in level 3–7 urbanization areas. The July effect was observed in patients receiving treatments at regional hospitals, public hospitals, and hospitals located in level 3–7 urbanization areas; however, the total cost of initial hospitalization of non-July admissions was higher than that for July admissions.

The weekend effect was observed in the total length of initial hospitalization, for which the length for weekend admissions was longer than that for non-weekend admissions. The length of initial hospitalization for weekend admissions was substantially longer than that for non-weekend admissions in regional, public, and private hospitals. The total hospitalization length for non-July weekend admissions was longer than that for non-July weekday admissions. Weekend admissions did not influence the health care quality but extended the total length of initial hospitalization. However, the influences for weekend admissions on the total cost of initial hospitalization were less significant.

## 6. Limitations

This study analyzed data from the NHIRD. However, because of its data limitations, we could not acquire data related to patients’ disease severity, total out-of-pocket medical expense amount, and clinical health care quality. The research used a complete dataset only ranging from 2006 to 2012, and it is impossible to infer long-term change.

Consequently, some factors (e.g., severity of illness and family support) may not have been included in the controls or statistical analysis model. Hence, the effects of these factors could not be discussed or inferred. This study performed a stratified analysis on patients with stroke only and could therefore make inferences regarding some patients rather than all patients covered under the NHI.

## Figures and Tables

**Table 1:** Basic characteristics of patients with stroke.

Variable	Number of Patients	Percentage (%)
Total number of patients	24,357	100.00
Patient characteristics		
Sex		
Female	9764	40.09
Male	14,593	59.91
Age (year)		
18–44	1328	5.45
45–54	3068	12.60
55–64	5012	20.58
65–74	6357	26.10
≥75	8592	35.28
Urbanization Level of Residential Area		
1	5923	24.32
2	7162	29.40
3	4043	16.60
4	3873	15.90
5	824	3.38
6	1410	5.79
7	1122	4.61
Insured Salary (NT$)		
≤17,280	7163	29.41
17,281–22,800	10,445	42.88
22,801–28,800	1544	6.34
28,801–36,300	1565	6.43
36,301–45,800	1712	7.03
45,801–57,800	715	2.94
≥57,801	1213	4.98
Charlson Comorbidity Index		
0	13,360	54.85
1–3	8807	36.16
4–6	1581	6.49
≥7	609	2.50
Whether Treated with Surgery		
Yes	16,467	67.61
No	7890	32.39
Hospital Characteristics		
Hospital Levels		
Medical center	9819	40.31
Regional hospital	14,538	59.69
Hospital Ownership		
Public hospital	6831	28.05
Private hospital	17,526	71.95
Urbanization Levels of Hospital Location		
1	8067	33.12
2	11,348	46.59
3	1461	6.00
4	3251	13.35
5	0	0.00
6	195	0.80
7	35	0.14
Total number of stroke patients treated per year (*n*)		
High patient number (>75th percentile)	20,096	82.51
Medium patient number (25th–75th percentile)	4252	17.46
Low patient number (<25th percentile)	9	0.04
Total number of neurologists and neurosurgeons (M ± SD)	15.14 ^b^	14.59 ^c^
Specialist characteristics		
Number of patients treated per year ^a^		
High patient number (>75th percentile)	15,115	62.06
Medium patient number (≤75th percentile)	9242	37.94
July/non-July admission		
July	2016	8.28
Non-July	22,341	91.72
Weekend/non-weekend admission		
Weekend	6920	28.41
Non-weekend	17,437	71.59
Hospital Admission Time		
July weekday admissions (July effect)	4330	17.78
Non-July weekend admissions (weekend effect)	5248	21.55
July weekend admissions (combined influence of July effect and weekend effect)	1756	7.21
Non-July weekday admissions	13,023	53.47
Health Care Quality Indicators		
Mortality within 30 days after hospitalization		
Yes	1603	6.58
No	22,754	93.42
Readmission within 14 days after hospital discharge		
Yes	985	4.04
No	23,372	95.96
Health Care Utilization Indicators		
Total length of initial hospitalization (day; M ± SD)	14.19 ^b^	24.69 ^c^
Total cost of initial hospitalization (NT$; M ± SD)	93,319 ^b^	188,233 ^c^

^a^ Most patients with stroke received treatments at medical centers or regional hospitals; therefore, the total number of patients treated by specialists did not contain the low patient number group. ^b^ Mean (M). ^c^ Standard deviation (SD).

**Table 2:** Logistic regression analysis with GEE models for the July effect and weekend effect on mortality rate within 30 days after hospitalization or readmission rate within 14 days after hospital discharge in patients with stroke.

Variable	Health Care Quality Indicators							
	Mortality within 30 Days after Hospitalization				Readmission within 14 Days after Hospital Discharge			
	OR	95% CI		*p* Value	OR	95% CI		*p* Value
July Effect and Weekend Effect								
Non-July weekday admissions (ref.)								
July weekday admissions (July effect)	0.93	0.79	1.09	0.348	1.08	0.90	1.31	0.407
Non-July weekend admissions (weekend effect)	1.04	0.64	1.68	0.884	0.96	0.55	1.70	0.900
July weekend admissions (combined influence of July effect and weekend effect)	0.88	0.52	1.47	0.622	0.98	0.54	1.80	0.952
Patient characteristics								
Sex								
Female (ref.)								
Male	0.94	0.84	1.04	0.234	0.99	0.87	1.13	0.882
Age (year)								
18–44 (ref.)								
45–54	0.77	0.61	0.99	0.038	0.79	0.59	1.05	0.106
55–64	0.58	0.46	0.74	<0.001	0.78	0.59	1.02	0.072
65–74	0.54	0.43	0.67	<0.001	0.65	0.49	0.85	0.002
≥75	0.88	0.70	1.09	0.226	0.61	0.47	0.79	<0.001
Urbanization Levels of Hospital Location								
7 (ref.)								
6	1.29	0.94	1.77	0.116	0.70	0.48	1.03	0.072
5	1.11	0.77	1.62	0.572	0.95	0.63	1.45	0.826
4	1.27	0.96	1.67	0.094	0.85	0.62	1.16	0.297
3	1.04	0.78	1.38	0.794	0.89	0.65	1.22	0.479
2	1.06	0.81	1.40	0.664	0.75	0.55	1.02	0.062
1	1.07	0.81	1.42	0.632	0.72	0.52	0.99	0.043
Insured Salary (NT$)								
≤17,280 (ref.)								
17,281–22,800	0.95	0.84	1.08	0.435	1.01	0.86	1.19	0.908
22,801–28,800	0.89	0.71	1.13	0.344	0.80	0.59	1.09	0.156
28,801–36,300	0.84	0.66	1.07	0.152	0.86	0.64	1.15	0.306
36,301–45,800	0.73	0.57	0.94	0.013	0.98	0.75	1.29	0.907
45,801–57,800	0.86	0.62	1.20	0.373	1.39	0.99	1.97	0.061
≥57,801	0.83	0.64	1.08	0.169	0.85	0.61	1.19	0.336
Charlson Comorbidity Index								
0 (ref.)								
1–3	1.21	1.08	1.36	0.001	1.09	0.95	1.25	0.242
4–6	2.34	1.97	2.78	<0.001	0.86	0.64	1.15	0.308
≥7	2.27	1.75	2.94	<0.001	1.25	0.85	1.84	0.258
Underwent Any Surgery								
No (ref.)								
Yes	3.74	3.20	4.37	<0.001	1.05	0.91	1.20	0.541
Specialist characteristics								
Number of patients treated per year								
Medium patient number (≤75th percentile) (ref.)								
High patient number (>75th percentile)	0.42	0.38	0.47	<0.001	0.83	0.73	0.95	0.007
Hospital Characteristics								
Hospital Levels								
Regional hospital (ref.)								
Medical center	1.01	0.87	1.17	0.936	0.88	0.73	1.06	0.178
Hospital Ownership								
Public hospital (ref.)								
Private hospital	0.90	0.80	1.01	0.072	1.15	0.98	1.34	0.084
Urbanization Levels of Hospital Location								
7 (ref.)								
6	6.46	0.82	50.59	0.076	0.88	0.23	3.44	0.858
5	-	-	-	-	-	-	-	-
4	2.08	0.28	15.58	0.476	0.63	0.19	2.12	0.456
3	2.09	0.28	15.72	0.476	0.62	0.18	2.11	0.441
2	2.00	0.27	14.97	0.498	0.67	0.20	2.26	0.523
1	2.11	0.28	15.79	0.468	0.68	0.20	2.31	0.539
Total number of stroke patients treated per year (*n*) ^a^								
Medium patient number (≤75th percentile) (ref.)								
High patient number (>75th percentile)	1.29	1.10	1.50	0.001	0.98	0.82	1.18	0.832
Total number of neurologists and neurosurgeons	0.99	0.98	0.99	<0.001	1.00	0.99	1.00	0.390
Admission year								
2006 (ref.)								
2007	1.05	0.88	1.26	0.580	1.20	0.93	1.55	0.163
2008	0.97	0.81	1.17	0.785	1.33	1.03	1.71	0.027
2009	0.98	0.82	1.18	0.857	1.34	1.04	1.73	0.023
2010	0.90	0.75	1.10	0.302	1.51	1.17	1.93	0.001
2011	0.96	0.80	1.16	0.706	1.71	1.34	2.18	<0.001
2012	0.94	0.76	1.16	0.559	2.05	1.60	2.64	<0.001

^a^ Most patients with stroke received treatments at medical centers or regional hospitals; therefore, the total number of patients treated by specialists did not contain the low patient number group.

**Table 3:** Multiple regression analysis with GEE models for the July effect and weekend effect on total length and cost of initial hospitalization in patients with stroke.

Variable	Health Care Utilization Indicators					
	Total Length of Initial Hospitalization			Total Cost of Initial Hospitalization		
	Coefficients	Standard Error	*p* Value	Coefficients	Standard Error	*p* Value
Intercept	6.88	1.39	<0.001	33,602.64	1.43	<0.001
July Effect and Weekend Effect						
Non-July weekday admissions (ref.)						
July weekday admissions (July effect)	0.98	1.02	0.316	0.98	1.02	0.386
Non-July weekend admissions (weekend effect)	1.08	1.05	0.125	0.95	1.06	0.352
July weekend admissions (combined influence of July effect and weekend effect)	1.06	1.06	0.319	0.91	1.06	0.110
Patient characteristics						
Sex						
Female (ref.)						
Male	0.95	1.01	<0.001	0.96	1.01	0.004
Age (year)						
18–44 (ref.)						
45–54	0.97	1.03	0.300	0.79	1.03	<0.001
55–64	0.96	1.03	0.110	0.74	1.03	<0.001
65–74	0.99	1.03	0.711	0.76	1.03	<0.001
≥75	1.09	1.03	0.002	0.84	1.03	<0.001
Urbanization Levels of Hospital Location						
7 (ref.)						
6	0.99	1.04	0.727	1.00	1.04	0.956
5	0.97	1.04	0.475	0.96	1.05	0.319
4	1.01	1.03	0.729	1.03	1.03	0.443
3	0.96	1.03	0.201	0.95	1.03	0.166
2	0.99	1.03	0.717	0.96	1.03	0.245
1	1.01	1.03	0.719	0.97	1.03	0.376
Insured Salary (NT$)						
≤17,280 (ref.)						
17,281–22,800	0.92	1.02	<0.001	0.92	1.02	<0.001
22,801–28,800	0.94	1.03	0.013	0.94	1.03	0.033
28,801–36,300	0.95	1.03	0.043	0.93	1.03	0.012
36,301–45,800	0.95	1.03	0.054	0.94	1.03	0.015
45,801–57,800	0.96	1.04	0.228	0.94	1.04	0.109
≥57,801	0.89	1.03	<0.001	0.94	1.03	0.060
Charlson Comorbidity Index						
0 (ref.)						
1–3	1.00	1.01	0.830	1.02	1.01	0.260
4–6	1.02	1.02	0.340	1.13	1.03	<0.001
≥7	1.04	1.04	0.348	1.13	1.04	0.003
Underwent Any Surgery						
No (ref.)						
Yes	1.49	1.01	<0.001	2.02	1.01	<0.001
Specialist characteristics						
Number of patients treated per year						
Medium patient number (≤75th percentile) (ref.)						
High patient number (>75th percentile)	0.71	1.01	<0.001	0.59	1.01	<0.001
Hospital Characteristics						
Hospital Levels						
Regional hospital (ref.)						
Medical center	1.07	1.02	<0.001	1.22	1.02	<0.001
Hospital Ownership						
Public hospital (ref.)						
Private hospital	0.96	1.01	0.001	1.05	1.02	0.001
Urbanization Levels of Hospital Location						
7 (ref.)						
6	0.63	1.19	0.008	0.85	1.20	0.363
5	-	-	-	-	-	-
4	0.90	1.17	0.492	0.99	1.19	0.948
3	0.88	1.17	0.409	1.10	1.19	0.563
2	0.92	1.17	0.617	1.05	1.18	0.764
1	0.97	1.17	0.837	1.11	1.19	0.533
Total number of stroke patients treated per year (*n*)						
Low patient number (<25 percentile) (ref.)						
High patient number (>75th percentile)	1.17	1.36	0.603	1.10	1.39	0.776
Medium patient number (25th–75th percentile)	1.36	1.36	0.317	1.35	1.39	0.367
Total number of neurologists and neurosurgeons	1.00	1.00	<0.001	1.00	1.00	0.001
Admission year						
2006 (ref.)						
2007	0.97	1.02	0.204	0.99	1.02	0.587
2008	0.97	1.02	0.099	1.02	1.02	0.418
2009	0.99	1.02	0.567	1.05	1.02	0.041
2010	0.98	1.02	0.420	1.09	1.02	0.000
2011	0.94	1.02	0.007	1.03	1.02	0.140
2012	0.96	1.02	0.094	1.05	1.03	0.042

**Table 4:** Stratified analysis: multiple regression analyses with GEE models for influence of July and weekend admissions on health care utilization indicators in patients with stroke receiving treatments at hospitals with different characteristics.

Hospital Characteristics ^a^	Variable	Health Care Utilization Indicators					
		Total Length of Initial Hospitalization			Total Cost of Initial Hospitalization		
		Coefficients	Standard Error	*p* Value	Coefficients	Standard Error	*p* Value
Medical Center							
	Non-July (ref.)	1.00			1.00		
	July	1.01	1.03	0.785	1.00	1.04	0.942
						
	Non-weekend (ref.)	1.00			1.00		
	Weekend	1.07	1.02	0.001 *	1.03	1.02	0.175
Regional Hospital							
	Non-July (ref.)	1.00			1.00		
	July	0.94	1.03	0.040 *	0.90	1.03	<0.001 *
						
	Non-weekend (ref.)	1.00			1.00		
	Weekend	1.06	1.02	0.001 *	1.02	1.02	0.285
Public Hospital							
	Non-July (ref.)	1.00			1.00		
	July	0.92	1.04	0.037 *	0.88	1.05	0.006 *
						
	Non-weekend (ref.)	1.00			1.00		
	Weekend	1.07	1.03	0.008 *	1.03	1.03	0.290
Private Hospital							
	Non-July (ref.)	1.00			1.00		
	July	1.00	1.03	0.961	0.97	1.03	0.235
						
	Non-weekend (ref.)	1.00			1.00		
	Weekend	1.06	1.02	0.000 *	1.02	1.02	0.144
Urbanization Level of Hospital Location 1–2							
	Non-July (ref.)	1.00			1.00		
	July	0.99	1.02	0.527	0.95	1.03	0.067
						
	Non-weekend (ref.)	1.00			1.00		
	Weekend	1.07	1.01	<0.001 *	1.03	1.02	0.032
Urbanization Level of Hospital Location 3–7							
	Non-July (ref.)	1.00			1.00		
	July	0.93	1.05	0.132	0.90	1.05	0.035 *
						
	Non-weekend (ref.)	1.00			1.00		
	Weekend	1.04	1.03	0.166	0.98	1.03	0.568

**^a^** The aforementioned stratified analysis models were all controlled for variables such as patient- and specialist-related characteristics. * *p* < 0.05.

**Table 5:** Stratified analysis: multiple regression analyses with GEE models for influence of different admission times on health care utilization indicators in patients with stroke receiving treatments at hospitals with different characteristics.

Hospital Characteristics ^a^	Variable	Health Care Utilization Indicators					
		Total Length of Initial Hospitalization			Total Cost of Initial Hospitalization		
		Coefficients	Standard Error	*p* Value	Coefficients	Standard Error	*p* Value
Medical Center	Non-July weekday (ref.)	1.00					
	July weekday (July effect)	0.96	1.03	0.138	-	-	-
	Non-July weekend (weekend effect)	1.07	1.02	0.007 *	-	-	-
	July weekend (combined effect)	1.05	1.04	0.180	-	-	-
Regional Hospital	Non-July weekday (ref.)	1.00					
	July weekday (July effect)	0.99	1.02	0.542	-	-	-
	Non-July weekend (weekend effect)	1.07	1.02	0.001 *	-	-	-
	July weekend (combined effect)	1.02	1.03	0.455	-	-	-
Public Hospital	Non-July weekday (ref.)	1.00					
	July weekday (July effect)	0.95	1.03	0.138	-	-	-
	Non-July weekend (weekend effect)	1.06	1.03	0.043 *	-	-	-
	July weekend (combined effect)	1.08	1.05	0.104	-	-	-
Private Hospital	Non-July weekday (ref.)	1.00					
	July weekday (July effect)	0.99	1.02	0.602	-	-	-
	Non-July weekend (weekend effect)	1.07	1.02	<0.001 *	-	-	-
	July weekend (combined effect)	1.02	1.03	0.419	-	-	-
Urbanization Level of Hospital Location 1–2	Non-July weekday (ref.)	1.00			1.00		
	July weekday (July effect)	0.97	1.02	0.134	0.96	1.02	0.044 *
	Non-July weekend (weekend effect)	1.07	1.02	<0.001 *	1.03	1.02	0.107
	July weekend (combined effect)	1.04	1.03	0.106	0.98	1.03	0.583
Urbanization Level of Hospital Location 3–7	Non-July weekday (ref.)	1.00			1.00		
	July weekday (July effect)	1.00	1.04	0.940	2.73	2.82	0.773
	Non-July weekend (weekend effect)	1.06	1.03	0.100	2.88	2.81	0.735
	July weekend (combined effect)	1.01	1.06	0.825	2.75	2.88	0.102

**^a^** The aforementioned stratified analysis models were all controlled for variables such as patient- and specialist-related characteristics. * *p* < 0.05.

## Data Availability

Data of this study were obtained from the National Health Insurance Research Databases released by the Ministry of Health and Welfare, Taiwan. These databases are not publicly available due to the Taiwan government’s legal restrictions of the Personal Information Protection Act. However, for conducting studies, researchers can apply for using these databases. If the proposals are approved, researchers can analyze data in the Health Data Science Center. Only de-identified data made in the tables or figures are allowed to be brought out from the center. These restrictions prohibit researchers from making the data publicly available.

## Abbreviations

NHI: National Health Insurance; NHIRD: National health insurance Research Database; ICD-9-CM: International Classification of Diseases, Ninth Revision, Clinical Modification; CI: confidence interval; ICU: intensive care units; and AMI: acute myocardial infarction.
